# Climate Food and Associations in Forming Races

**Published:** 1885-04

**Authors:** James Truman

**Affiliations:** Philadelphia, Pa.


ARTICLE IV.

### CLIMATE, FOOD, AND ASSOCIATIONS IN FORMING RACES.

BY JAMES TRUMAN, D. D. S., PHILADELPHIA, PA.

The prevalent idea that teeth vary according to climate, food, etc., is probably true, and needs no argument to refute it; but to what extent this variation takes place in teeth, more than in other tissues, is not clearly indicated. In fact, it is a subject encompassed with difficulties. Dentistry is too young, and the trained workers too few, and the statistics, collected from professional sources, too meager to arrive at any definite conclusions. It is, therefore, only, possible to skim over the surface, and, perhaps, glean a few facts, leaving the result to the future for that correction which must necessarily come from more positive observation. That climatic influences have largely to do with the formation of tissue is axiomatic, and would seem to need no words to make it self-evident, but the processes through which this is effected are by no means clear. They enter into the realms of mysterious influences that operate to evolve life from the minutest atom to the fully-developed animal; from the unorganized protoplasm to the almost God-like mentality of man. Till we can define what the correlation of forces really means, till we can fathom the mysterious border-land, which no microscope has ever penetrated, we must grope in profound darkness. Here observation ends and theory begins, and yet, behind this theory, in the illimitable void, lies the true home of the forces, which aggregated, create life and life-forms where-ever we find them. The most powerful forces are the intangible. We call the fall of bodies to the earth the force of gravitation. We recognize the fact that the universe is held in position by a power invisible and incomprehensible to the human intellect. The world of causation lies beyond our present reasoning powers or our most powerful objectives. We recognize certain effects, but the causes are shadowed in the realms of mysterious life.

Whether we view life in the concrete or examine it in its simplest forms, we are impressed with the fact that the laws of formation are everywhere imperiously present, and that atoms, organized or unorganized, are but the creatures of influences which through all apparent aberrations, are resolved finally into harmonious relations. While we may formulate the law into words and say, evolution is progressive development, we are met at the begining of our inquiry by apparent contradictions—contradictions which the few thousand years accorded us for observation have failed to clear up. The apparently unchanging color of the different races of men, and their typical forms, continuing through successive ages, have never been satisfactorily traced to climatic influences or differences in food and surroundings. The world has rested content with the explanation that the color of the negro was the result of tropical heat through countless generations, but it is somewhat difficult to reconcile this statement with the facts of history and observation. The effect of color was supposed to be largely dependent on the degree of distance from equatorial regions. This theory is met by the equally difficult fact of the existence of light-shaded men in the interior of Africa, and from that other long and well known fact that the coppery tinge is common to races in temperate zones. The negro appears to be a distinct type and cannot be confounded with any of the other tropical races. It may be argued, with some degree of plausibility, that if he is the result of climatic influences, then, by the same influences, other races, similarly circumstanced in regard to heat, should have been similarly formed; but they are wanting in many of the attributes of color, form, etc. The North American Indian indicates by his color his probable Asiatic origin, for there is nothing in climate to cause the tinge, else would two hundred and more years have given some evidence of change in our own race. But if change exist at all, it has been on the side of improvement, and the American of to-day—in the North, has less color than the races from which he sprang. Yet, the negro of the present is the negro of all history. When Assyria was in its glory he was a factor in its civilization, and lives to-day in its ruined temples. When Egypt was young, Africa was evidently old, and the Nubian of the nineteenth century is the Nubian of a period of which only the records in stone are left. We can recognize this coloring of races from the negro to the pure Caucasian, but we are left stranded on the shoals of inconclusive data, in bays to which no rivers lead, in results which, however closely followed, are traceable to no absolute cause. If climate produced in uncounted ages a negro, it should in similiar periods undo its work under more favorable circumstances. The records of observation are too limited to solve this problem. So difficult is it to reconcile facts with the theory of evolution, that men like Agassiz have not been able to accept it, but have clung—perhaps unreasonably—to the old and equally difficult theory of distinct creations. While admitting the force of the argument that no perceptible change has occurred in history, written or unwritten, in the color of man as regard races, we do know that the color deepens by exposure to the tropical suns; and this, through successive action of generations, ought to produce a result as intense as that of the negro. There should be some evidence of value in its respect found in the Spanish races that have peopled the tropical regions of this continent, but, unfortunately, they are of too mixed a character to be worth anything as a basis for inductive reasoning.

Without entering into this line of argument further, it is very clear that the surroundings to which animals and men have been subjected have had much, if not all, to do with the various productions that we witness. Probably no instance on as large a class in modern times has occurred as the changes that have taken place in our own country. The time is short—barely more than two centuries—and yet the American type is as fixed and as marked as any of the older races of the world. It is said, and with truth, that we are a mixed race; but making due allowance for this, there is a certain undefined and undefinable appearance that marks our race, as it marks all races. The American form is as distinct from the German stock, from which it in the main sprung, as it is possible to imagine. Who can fail to observe the square shoulders and broad hips of the Teuton and not draw comparisons favorable to the sloping shoulders and graceful lines of the form of the American-born? These are marked distinctions and strike the eye at once. They demonstrate what I regard as a fact, that slowly, but surely, we are growing a type of men and women that in time will be a distinct race, grafted on old stocks, but molded by climate, food, natural associations and the law of harmonious development into a class—whether superior or inferior, at least widely different from all others.—[Trans. Odontological Society.] *Items of Interest.*

